# Interaction between birth characteristics and *CRHR1, MC2R, NR3C1, GLCCI1* variants in the childhood lymphoblastic leukemia risk

**DOI:** 10.3389/fonc.2023.1274131

**Published:** 2024-01-29

**Authors:** Vitoria Müller de Carvalho, Alython Araujo Chung-Filho, Flávio Henrique Paraguassu Braga, Paulo Chagas-Neto, Sheila Coelho Soares-Lima, Maria S. Pombo-de-Oliveira

**Affiliations:** ^1^ Research Center, Molecular Carcinogenesis Program, Instituto Nacional de Câncer (INCA), Rio de Janeiro, Brazil; ^2^ National Placental and Umbilical Cord Blood Bank, Instituto Nacional de Câncer (INCA), Ministério da Saúde (MS), Rio de Janeiro, Brazil

**Keywords:** *MC2R* rs1893219 A>G, *GLCCI1* rs37972 C>T, acute lymphoblastic leukemia, interaction, Brazil

## Abstract

**Background:**

The incidence rate of childhood acute lymphoblastic leukemia (ALL) differs worldwide, and the interplay between hemostasis actors and the maladaptive responses to environmental exposures has been explored. It has been proposed that endogenous cortisol, induced by different triggers, would eliminate pre-leukemic clones originated *in utero*. Herein, we tested if the interaction between *CRHR1rs242941 C>A, MC2Rrs1893219 A>G, NR3C1rs41423247 G>C*, and *GLCCI1rs37972 C>T* (players in glucocorticoid secretion) and birth characteristics would be associated with ALL risk.

**Methods:**

Children aged <10 years were enrolled within the EMiLI project (period: 2012 to 2020). The study had three steps: (1) observational analysis of birth characteristics (*n* = 533 cases and 1,603 controls); (2) genotyping to identify single-nucleotide variants (*n* = 756 cases and 431 controls); and (3) case-only to test gene–environment interactions (*n* = 402 cases). Genetic syndromes were exclusion criteria. The controls were healthy children. The distribution of the variables was assessed through Pearson’s chi-square test. Logistic regression (LR) tests were run fitted and adjusted for selected covariate models to estimate the association risk. Formal interaction analysis was also performed. Genotyping was tested by qPCR with TaqMan probes (*NR3C1*) or by high-resolution melting (*MC2R* and *GLCCI1*). Hardy–Weinberg equilibrium (HWE) was accessed by the chi-square test. The genotype–risk association was tested in co-dominant, dominant, and recessive models. The gene–environment interaction odds ratio (iOR) was assessed in case-only.

**Results:**

Low birthweight, C-section, and low maternal schooling were associated with increased risk for ALL, adjOR 2.11, 95% CI, 1.02–4.33; adjOR 1.59, 95% CI, 1.16–2.17; and adjOR 3.78, 95% CI, 2.47–5.83, respectively, in a multiple logistic regression model. *MC2R* rs1893219 A>G was negatively associated with ALL (AG: OR = 0.68; 95% CI = 0.50–0.94 and GG: OR = 0.60; 95% CI = 0.42–0.85), while for *GLCCI1* rs37972 C>T, TT was positively associated with ALL (OR = 1.91; 95% CI = 1.21–3.00). The combination of genotypes for *MC2R* (AA) and *GLCCI1* (TT) increased ALL risk (OR = 2.61; 95% CI = 1.16–5.87). In a multiplicative interaction, *MC2R* rs1893219 A>G was associated with children whose mothers had less than 9 years of schooling (iOR = 1.99; 95% CI = 1.11–1.55).

**Conclusion:**

Our study has demonstrated a significant association between *MC2R* rs1893219 A>G (reduced risk) and *GLCCI1* rs37972 C>T variants (increased risk) and childhood ALL susceptibility. Based on this evidence, genes controlling the HPA axis activity may play a role in leukemogenesis, and further investigation is needed to substantiate our findings.

## 1 Introduction

The causal mechanism for leukemia gathers environmental factors, inherited susceptibility from polygenic variants, and chances of interactions. Models for interplay of such chances have been proposed for childhood acute lymphoblastic leukemia (ALL) mainly concerning B-cell precursor-ALL (Bcp-ALL), in which chromosomal translocations can arise *in utero* (1–3). Greaves and collaborators have unraveled the clonal evolution of Bcp-ALL endorsing the model of multiple gene–environment factors in the causal mechanisms of ALL pathogenesis (4–6). One of the pillars supporting the Greaves hypothesis is the epidemiological association of birth characteristics, the first year of the child’s life hygiene and socialization, as well as immune responses to early infections (3, 7). This biological evidence has opened avenues to the so-called “adrenal hypothesis” that has also emerged from observational studies of global differences on ALL incidence rate and environmental exposures in less developed societies (8). These differences led to the speculation that the hypothalamic–pituitary–adrenal (HPA) axis decreases the kinetics of disappearance of pre-leukemic clone through qualitative and quantitative plasma cortisol levels in populations with deprivation and infection exposures (8). Differences in the reactivity of the HPA axis have been proposed at genomic levels and its setting occurs during the intrauterine life, although it can also be reset by stressful experiences in early life or chronic stress as individuals go through threatening experiences (9, 10).

Physiologically, cortisol and cortisone, the so-called endogenous glucocorticoids (GCs), are released according to a cascade of interacting signals and actions from different organs of the HPA axis, initiated by corticotrophin-releasing hormone (CRH). CRH acts through the interaction with the CRHR1 receptor in the hypothalamus, leading to the release of the adrenocorticotropic hormone (ACTH). In the adrenal cortex, ACTH binds its receptor MC2R (melanocortin type 2 receptor), stimulating cortisol secretion. Cortisol will ultimately bind to its receptor NR3C1 (receptor nuclear subfamily 3, group C, member 1) in the target cell and stimulate the expression of target genes (11, 12). Glucocorticoid Induced 1 (*GLICCI1*) is one of these genes and, although its functions are not completely elucidated, it seems to both modulate glucocorticoid efficiency and regulate apoptosis (13). Based on their key functions in the HPA axis as well as on our interest of evaluating the different steps in this pathway, *CRHR1, MC2R, NR3C1*, and *GLICCI1* were selected to evaluate the possible contribution of genetic variants to the variability in GC secretion levels. The specific variants were selected based on their frequencies and on previous studies suggesting their association with GC-associated outcomes (14–16).

In addition, we have recently described the association between being born through Cesarean-section (C-S), being the first child, and low birth weight (LBW) with increased risk for ALL, although the biological plausibility remains speculative and deserve further studies (17). Researchers claim that mode of delivery through C-S and children with LBW would present low epinephrine, cortisol, and cytokine levels, elements necessary to establish a healthy microbiome and a harmonic immune system (10). Herein, we hypothesized that gene variants may impact the GCs’ endogenous levels, subsequently allowing the expansion of possible leukemic clone that arose *in utero*, contributing to ALL development. Therefore, the aim of the study was to investigate whether ALL risk is associated with child’s birth characteristics and genetic susceptibility involving the HPA axis.

## 2 Materials and methods

### 2.1 Study design and subjects

Children with less than 10 years of age at diagnosis of Bcp-ALL and T-ALL, from a hospital-based case–control study, were included over the period of 2012 to 2020. The present study was part of the project “Epidemiology of Multi-institutional Study Group of Acute Leukemia (EMiLI)”, for which the enrollment of cases and controls was recently fully described (17). Herein, the analysis was carried out in three steps:

(1) Case–control study (*n* = 2136) to revisit the birth characteristic’s risk estimate adjusted by maternal schooling. The variables for the models included leukemia subtypes (484 Bcp-ALL and 49 T-ALL), race/ethnicity, child sex, mode of child delivery, and birth weight, while maternal age at childbirth and maternal schooling were the covariates. The maternal schooling was assessed as a proxy for the child’s socioeconomic level.
(2) Genotyping study. Genomic DNA from cases (*n* = 756) and controls (*n* = 431) was obtained from peripheral blood cells, using the salting-out method (15). To identify *CRHR1 rs242942 C>T, MC2R rs1893219*, and *GLCCI1 rs37972 C>T* variants, polymerase chain reactions followed by high-resolution melting (HRM) were performed. The oligonucleotides were designed to each region of interest and the protocol conditions are shown in **Supplementary Table 1**. Approximately 10 to 15 samples genotyped for each variant were randomly selected to validate the HRM results using Sanger direct sequencing. The electropherogram analysis was performed using the Mutation Survey software (SoftGenetics, Pennsylvania, USA). *NR3C1* rs41423247 G>C allelic discrimination was performed using a TaqMan assay (C:86507873_10, Thermo Fisher) and TaqMan Genotyping Master MixTM (Thermo Fisher), in Rotor-gene 6000 (QIAGEN, Germany). The variables included in this step of the study were leukemia subtypes, racial/ethnicity, child sex, allele status, and genotype frequencies.
(3) Case-only. In this step, the impact of the multiplicative interaction between genotypes and environmental exposures on ALL risk was estimated (18). For this, cases with and without the risk allele were compared with respect to exposure (19). The validity of this design to estimate the interaction odds ratio (IOR) depends on the assumption that among controls, genotype and exposure are independent, i.e., the fact of having the gene variant will not influence the exposure variable.

### 2.2 Statistical analysis

The sample size was calculated considering controls per case (ALL subtypes; ratio: 1:3), α = 0.05, β = 0.2 for power = 0.80. To explore the impact of the birth characteristics (C-S and LBW) considering the hypothesis of the intrauterine origin of ALL and the peak incidence, we have stratified the cases into two age strata: (1) children who were up to 5 years of age and (2) children aged older than 6 up to 10 years at the diagnosis. The association between C-S and LBW and ALL risk was estimated using multiple logistic regression (MLR) adjusted for the mother’s age at childbirth [adjusted odds ratio (adjOR) and 95% confidence interval (95% CI)]. The core model with variables of interest consisted of mode of delivery (C-S vs. vaginal), child’s ethnicity (White versus multiracial, as reference), birth weight [ ≤ 2,500, 2,500 to 3,500 (reference); >3,550], gestational age (<37; ≥37 weeks), maternal ages [<25; 25–34 (reference); ≥35 years old], and maternal schooling (≤9, >9 years).

Genotyping: The genotype frequency distribution in controls was analyzed to test the Hardy–Weinberg equilibrium (HWE) by Pearson’s χ^2^ test; *p* > 0.05 was in accordance with HWE. Frequency differences between cases and controls were assessed through logistic regression test, the OR and 95% CI were calculated in the codominant (heterozygous versus wild type and variant homozygous versus wild type), dominant (variant homozygous + heterozygous versus wild type), and recessive (variant homozygous versus heterozygous + wild type) models. Variants associated with ALL risk were also analyzed for gene–gene interactions. In the additive model, the relative excess risk due to interaction (RERI), attributable proportion (AP), and synergy index (SI) were measured. These values and their respective delta-method 95% CI were calculated as published by Anderson et al. (20).

Case-only: We used unconditional logistic regression to calculate the IOR 95% CI controlled by the child’s ethnicity (Whites as reference). Only genetic variants associated with ALL risk in step 2 were included in the model, and the non-risk alleles were used as reference. The risk factors analyzed included the mode of delivery (vaginal as reference), birth weight (2,500–3,499 as reference), and mother education (>9 years as reference). All statistical analyses were performed using R studio version R4.1.1 and IBM SPSS Statistics version 26.

Ethical aspects: All collaborating Brazilian institutions approved the study and written informed consents were obtained from mothers or relatives responsible for the enrolled children. The Ethics and Scientific Committees of Instituto Nacional de Cancer approved this study (INCA/CAEE #626.268; CEP/CONEP 1.394.043).

## 3 Results

### 3.1 Birth characteristics

In this study, a total of 2,136 children, including 533 ALL cases and 1,603 controls, were included in the initial phase. The case:control ratio was 1:3. Among cases, 484 (90.8%) were Bcp-ALL and 49 (9.2%) were T-ALL (**Table 1**). Most cases were children up to 5 years of age at diagnosis (mean age: 3.5 years). Missing information (MI) was less than 4.7% for both cases and controls in the variables of interest, notably mode of delivery, birth weight, and maternal age. Cases’ mothers have reported lower schooling than controls (*p* = 0.001). The proportion of mode of delivery was similar between the groups, while LBW (≤2,500) was more prevalent among ALL cases (*p* = 0.001).

**Table 1:** Distribution of variable frequencies of 533 acute lymphoblastic leukemia and their 1603 controls.

Variables	Total N, 2136 (%)	Cases N, 533 (%)	Controls N, 1603 (%)	*p* value
Leukemia Subtypes				
BCP-ALL	–	484 (90.8)	–	
T-ALL	–	49 (9.2)	–	
Child's age (years)				0.001
0-5	1570 (73.5)	431 (80.9)	1139 (71.1)	
≥ 6	566 (26.5)	102 (19.1)	464 (28.9)	
*Mean (min-max) SD*	3.7 (0.0-10) 2.8	3.5 (0.0-10) 2.5	3.0 (0.0-10) 2.9	
Sex				0.85
Females	950 (44.5)	239 (44.8)	711 (44.4)	
Males	1186 (55.5)	294 (55.2)	892 (55.6)	
Child´s Ethnicity				0.99
Whites	1058 (49.5)	264 (49.5)	794 (49.5)	
Multiracial	1078 (50.5)	269 (50.5)	809 (50.5)	
Mode of delivery				0.60
Vaginal	976 (45.7)	242 (45.4)	734 (45.8)	
C-Section	1157 (54.2)	291 (54.6)	866 (54.0)	
MI	3 (0.1)	0 (0.0)	3 (0.2)	
Birth Weight (grams)				0.001
≤ 2500	87 (4.1)	40 (7.5)	47 (2.9)	
2501-3500	1246 (58.3)	323 (60.6)	923 (57.6)	
> 3500	777 (36.4)	145 (27.2)	632 (39.4)	
MI	26 (1.2)	25 (4.7)	1 (0.1)	
*Mean (min-max) SD*	3362(1153-6125) 492,1	3250(1153-5200) 523,6	3398(1805-6125)473,2	
Maternal Schooling (years)				0.001
< 9	223 (10.4)	139 (26.1)	84 (5.2)	
≥ 9	1413 (66.1)	375 (70.4)	1038 (64.8)	
MI	500 (23.4)	19 (3.5)	279 (17.4)	
*Mean (min-max) SD*	10.7 (0.0-26) 3.48	10.9 (0.0-26) 4.29	10.6 (0.0-25) 3.03	
Maternal age				0.001
< 25	783 (36.7)	228 (42.8)	555 (34.6)	
25-34	1009 (47.2)	228 (42.8)	781 (48.7)	
≥ 35	325 (15.2)	64 (12.0)	261 (16.3)	
MI	19 (0.9)	13 (2.4)	6 (0.4)	
*Mean (min-max) SD*	27.1 (13.0-51.0) 6.2	26.3 (13.0-43.0) 6.8	27.0(18.0-51.0) 6.1	

BCP-ALL, B-cell acute lymphoblastic leukemia; T-ALL, T-cell Lymphoblastic Leukemia; N, number; MI, missing information; SD, standard deviation; Min, minimum; max, maximum.

Brazil, 2012-2020.

In the multiple logistic regression model (**Table 2**), both in the crude analysis and after adjustment by maternal age, LBW, C-section, and low maternal schooling were associated with increased risk for ALL, adjOR 2.11, 95% CI, 1.02–4.33; adjOR 1.59 95% CI, 1.16–2.17; and adjOR 3.78, 95% CI, 2.47–5.83, respectively.

**Table 2:** Multiple logistic regression-derived odds ratio for childhood acute lymphoblastic leukemia and their controls and environmental exposure.

Variables	ALL *N* = 492 (%)	Controls *N* = 1,106 (%)	Crude OR 95% CI	*p-*value	adj OR 95%CI	*p-*value
Child’s ethnicity						
White	250 (50.8)	559 (50.5)	1.00*		1.00*	
Multiracial	242 (49.2)	547 (49.5)	0.91 (0.73–1.13)	0.400	1.11 (0.82–1.50)	0.514
Birth weight (g)						
≤2,500	36 (7.3)	24 (2.2)	2.87 (1.64–4.91)	0.001	2.11 (1.02–4.33)	0.044
2,501–3,500	313 (63.6)	635 (57.4)	1.00*		1.00*	
>3,550	143 (29.1)	447 (40.4)	0.65 (0.51–0.83)	0.001	0.45 (0.32–0.65)	0.002
Mode of delivery						
Vaginal	226 (45.9)	547 (49.5)	1.00*		1.00*	0.004
C-section	266 (54.1)	559 (50.5)	1.14 (0.92–1.43)	0.230	1.59 (1.16–2.17)	
Maternal schooling (years)						
<9	129 (26.2)	84 (7.6)	4.22 (3.12–5.72)	0.001	3.78 (2.47–5.83)	0.001
≥9 (ref)	363 (73.8)	1,022 (92.4)	1.00*		1.00*	

All variables are in the model; Unconditional analysis; C-section and birthweight adjusted by maternal age, ALL, acute lymphoblastic leukemia; *, reference.

Brazil, 2012–2020. g, grams; n, number; OR, odds ratio; C-section, Cesarean section; 1.00*, reference group.

### 3.2 Genotyping

A total of 756 cases and 431 controls were genotyped for *CRHR1rs242941 C>A, MC2Rrs1893219 A>G, NR3C1rs41423247 G>C*, and *GLCCI1rs37972 C>T* variants, using only samples of optimal DNA quality (**Table 3**). There was no statistically significant difference between cases and controls regarding the variables included in the models. The controls’ genotypic frequencies for all variants were in HWE. A null result was found regarding the variants *CRHR1*rs242941 C>A and *NR3C1*rs41423247 G>C in all models assessed, while *MC2R* rs1893219 A>G and *GLCCI1* rs37972 C>T were associated with genetic predisposition to ALL development (**Table 4**). *MC2R* rs1893219 A>G showed an inverse association in models adjusted by race (DM: adjOR, 0.65, 95% CI, 0.48–0.87), demonstrating a significant protective role, while *GLCCI1 rs37972 C>T* was positively associated in the recessive model (RM) (adjOR, 1. 64, 95% CI, 1.11–2.43). The analysis according to race, sex, ALL subtypes, and age strata are shown in the **Supplementary Material**. Among white children, the *MC2Rrs*1893219 A>G variant was negatively associated with ALL in the dominant model (DM) (OR, 0.58, 95% CI, 0.38–0.88) while *GLCCI1rs37972 C>T* was positively associated in the RM (OR, 1.83, 95% CI, 1.01–3.34) (**Supplementary Table 2**). The same risk association pattern was found in Bcp-ALL and T-ALL subtypes (**Supplementary Table 3**) and among male children (**Supplementary Table 4**).

**Table 3:** Main characteristics and frequencies of gene variants of acute lymphoblastic leukemia and controls.

Variables		ALL *N* = 756 (%)	Controls *N* = 431 (%)	*p*-value
Sex				
	Male	428 (56.6)	233 (54.1)	0.39
	Female	328 (43.4)	198 (45.9)	
Race/skin color				
	Multiracial	407 (53.8)	240 (55.7)	0.53
	White	349 (46.2)	191 (44.3)	
ALL subtypes				
	BCP-ALL	654 (86.5)	–	–
	T-ALL	102 (13.5)	–	
Variant frequencies*				
	*CRHR1 rs242941* C>A	0.38	0.40	–
	*MC2R rs1893219* A>G	0.48	0.55	
	*NR3C1 rs41423247* G>C	0.23	0.23	
	*GLCCI1 rs37972* C>T	0.37	0.32	

N, number; ALL, acute lymphoblastic leukemia includes T-cell ALL and B-cell precursor ALL; NA, not applicable; (*) the Hardy–Weinberg equilibrium was tested for CRHR1, MC2R, NR3C1, and GLCCI1. p-values = 0.70, 0.50, 0.18, and 0.50, respectively, among the control group.

Brazil, 2012–2020.

**Table 4:** Gene variants and the genetic predisposition risks in acute lymphoblastic leukemia.

Genotype	Cases *n* (%)	Controls *n* (%)	Crude OR (95% CI)	AdjOR (95% CI)	*p-*value
***CRHR1*** ***r*s242941 C>A**	683 (100)	379 (100)			
CC	264 (38.7)	136 (35.9)	1.0*		
CA	315 (46.1)	185 (48.8)	0.88 (0.67–1.16)	0.89 (0.68–1.18)	0.42
AA	104 (15.2)	58 (15.3)	0.92 (0.63–1.35)	0.80 (0.56–1.14)	0.22
DM			0.89 (0.68–1.15)	0.94 (0.64–1.37)	0.74
RM			0.99 (0.70–1.41)	1.01 (0.71–1.43)	0.97
***MC2R*** **rs1893219 A>G**	608 (100)	401 (100)			
AA	180 (29.6)	85 (21.2)	1.0*		
AG	275 (45.2)	192 (47.9)	0.68 (0.49–0.93)	0.68 (0.50–0.94)	0.02
GG	153 (25.2)	124 (30.9)	0.58 (0.41–0.83)	0.60 (0.42–0.85)	<0.001
DM			0.64 (0.48–0.86)	0.65 (0.48–0.87)	<0.001
RM			0.75 (0.57–0.99)	0.76 (0.57–1.00)	0.05
***NR3C1*** **rs41423247 G>C**	693 (100)	407 (100)			
GG	410 (59,2)	244 (60.0)	1.0*		
GC	243 (35,1)	136 (33.4)	1.06 (0.82–1.38)	1.05 (0.81–1.37)	0.71
CC	40 (5,8)	27 (6.6)	0.88 (0.53–1.47)	0.88 (0.52–1.47)	0.62
DM			1.03 (0.81–1.33)	1.02 (0.80–1.31)	0.86
RM			0.86 (0.52–1.43)	0.85 (0.51–1.41)	0.52
***GLCCI1*** **rs37972 C>T**	678 (100)	410 (100)			
CC	273 (40.3)	187 (45.6)	1.0*		
CT	305 (45.0)	184 (44.9)	1.14 (0.87–1.47)	1.13 (0.87–1.47)	0.35
TT	100 (14.7)	39 (9.5)	1.76 (1.16–2.66)	1.75 (1.16–2.65)	0.01
DM			1.24 (0.97–1.59)	1.24 (0.97–1.59)	0.09
RM			1.65 (1.11–2.44)	1.64 (1.11–2.43)	0.01

n, number; OR, odds ratio; 95% CI, 95% confidence interval; RM, recessive model; DM, dominant model; AdjOR, adjusted odds ratio by race. *, reference group.

**Figure 1:**
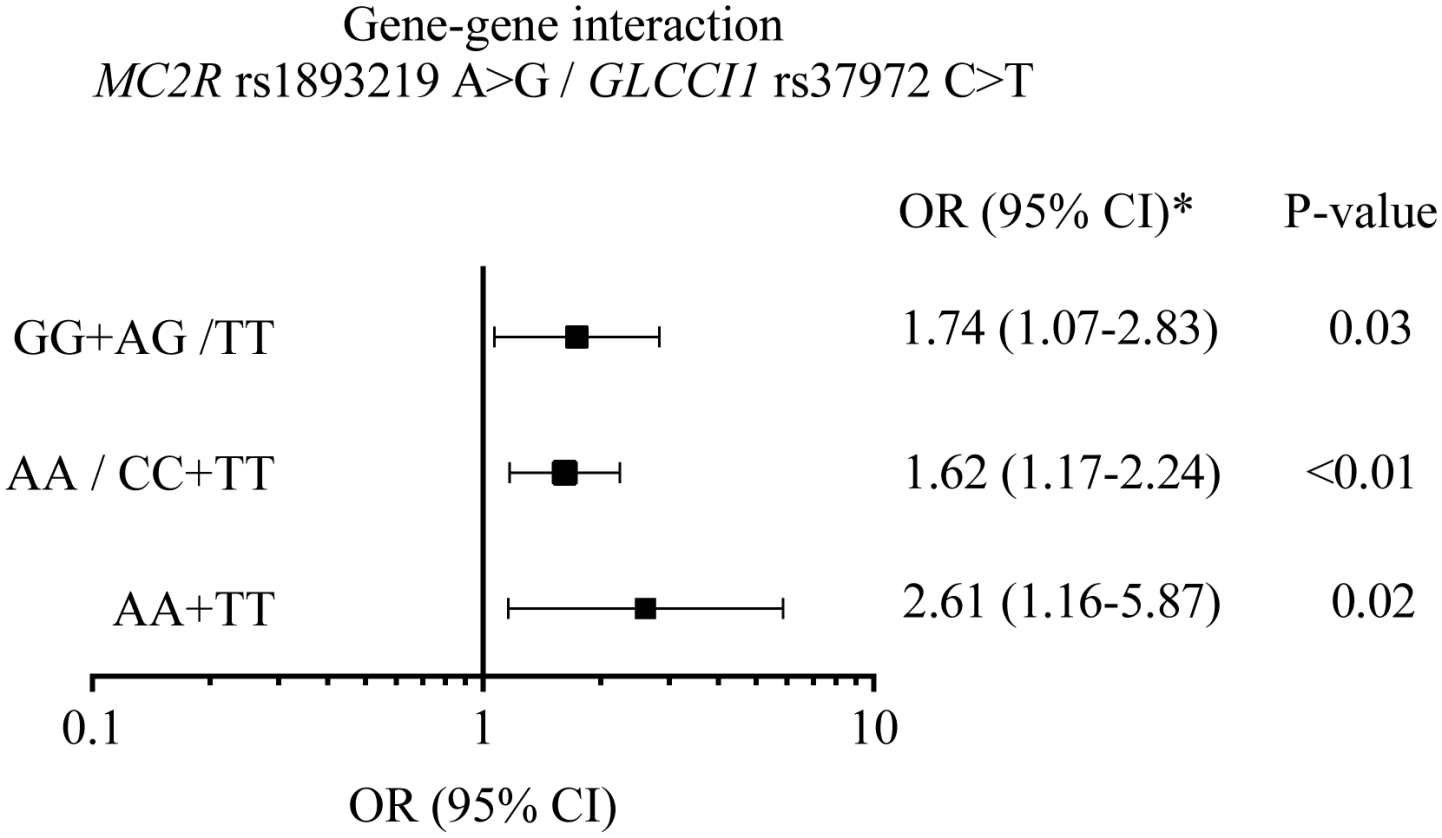
A graphic plot summarizing the effect of the interaction of *MC2R* rs1893219 and *GLCCI1* rs37972 variants in acute lymphoblastic leukemia risk. The association between genotypic combinations of the *MC2R* rs1893219 A>G and *GLCCI1* rs37972 C>T variants tested with adjusted odd ratio (adjOR) 95% confidence interval (CI) by race; GG+AG/TT, genotype of protection risk; AA/CC+TT, genotype of protection risk, AA+TT, genotype of high risk. The relative excess risk in the additive model was RERI=0.26 (-2.01-2.54), AP, attributable proportion=0.10 (-0.70-0.90) and SI, synergy index=1.19(0.7-5.19) (20).

The combination of wild-type homozygous genotype for *MC2R* (AA) and variant homozygous genotype for *GLCCI1* (TT), risk genotypes for the respective genes, showed a stronger association with ALL if compared to the individual effect of each variant (OR = 2.61; 95% CI = 1.16–5.87). The sum of the interaction analysis shown in **Figure 1**, however, revealed no synergistic or inhibitory effect between the variants (RERI, 0.26; 95% CI, −2.01–2.54).

### 3.3 Epidemiologic–genotyping assessment

Finally, the effect of *MC2R* rs1893219 A>G AA and *GLCCI1* rs37972 C>T was tested for interactions with birth weight, mode of delivery, and maternal education (**Table 5**). The model adjusted by race did not show interaction with *GLCCI1* rs37972 C>T risk, although an estimate risk factor was attributed to children with mothers of less than 9 years of schooling (iOR, 1.86, 95% CI = 1.06–3.26; Adj OR, 1.99; 95% CI = 1.11–3.55).

**Table 5:** Case-only analysis of gene–environment interaction for *MC2R* rs1893219 A>G and *GLCCI1* rs37972 C>T.

Variable	*MC2R* rs1893219 A>G *n* = 323					*GLCCI1* rs37972 C>T *n* = 348				
	AA, *n* (%)	AG+GG, *n* (%)	iOR (95% CI)	AjiOR	*p*-value	TT, *n* (%)	CC+CT, *n* (%)	iOR (95% CI)	AjiOR	*p*-value
Birth weight (g)										
2,501–3,500	53 (66.3)	113 (57.1)	1.0*	1.0*		30 (62.5)	172 (62.5)	1.0*	1.0*	
≤2,500	9 (11.2)	11 (5.5)	2.05 (0.80–5.24)	2.15 (0.84–5.54)	0.11	3 (6.3)	22 (8.0)	0.78 (0.22–2.78)	0.78 (0.22–2.77)	0.70
>3,500	18 (22.5)	74 (37.4)	0.61 (0.33–1.12)	0.59 (0.32–1.09)	0.09	15 (31.2)	81 (29.5)	1.06 (0.54–2.08)	1.05 (0.54–2.07)	0.88
Mode of delivery										
Vaginal	44 (49.4)	97 (41.5)	1.0*	1.0*		20 (39.2)	132 (44.4)	1.0*		
C-Section	45 (50.6)	137 (58.5)	0.72 (0.44–1.18)	0.72 (0.44–1.18)	0.20	31 (60.8)	165 (55.6)	1.24 (0.68–2.28)	1.23 (0.67–2.26)	0.50
Maternal education										
≥9 years	56 (67.5)	177 (79.4)	1.0*	1.0*		38 (76.0)	207 (74.2)	1.0*	1.0*	
<9 years	27 (32.5)	46 (20.6)	1.86 (1.06–3.26)	1.99 (1.11–3.55)	0.02	12 (24.0)	72 (25.8)	0.91 (0.45–1.83)	0.93 (0.46–1.88)	0.83

n, number; iOR, interaction odds ratio; AjiOR, interaction odds ratio adjusted for race/ethnicity; 95% CI, 95% confidence interval.

Brazil, 2012–2020. *, reference group.

## 4 Discussion

This is the first study that explores the HPA axis and childhood ALL through the effect of germline variants and a gene–environment interaction study model (8, 19). In the observational assessment, LBW, C-S, and low maternal schooling were positively associated with ALL risk. Worldwide, low education level is a proxy for socioeconomic status, and it has been associated with the functioning of the maternal HPA axis during pregnancy, impacting the health of the fetus. In populations with low human development index (HDI), LBW was associated with higher maternal cortisol levels at pregnancy, in turn leading to an LBW offspring with severe GC secretion deficiency (21, 22). However, the impact of gene variants in the HPA axis function has not been fully assessed. Within the complexity of ALL multifactorial etiopathogenesis, a role for genetic susceptibility involving genes responsible for GCs’ secretion cannot be dismissed (8, 23, 24).

This study focused on *CRHR1, MC2R, NR3C1*, and *GLICCI1*, four genes within the HPA axis, and the association of their variants with C-S, birthweight, and ALL. *CRHR1* and *NR3C1* variants did not show any association with birth weight and ALL. This result is in line with Schneider et al. (2020), who have found no association between genetic variants in the GC receptor gene and LBW (25). However, *NR3C1* seems to be crucial to the induction of apoptosis mediated by GCs in lymphoblasts and its regulation is essential in ALL treatment. Therefore, the null results found here regarding the risk association with ALL do not exclude its relevance in investigations related to resistance to GC therapy.

MC2R, another key receptor in the HPA axis, is much less studied. The variant *MC2R* rs1893219 A>G, mapped to *MC2R* promoter region, is the most frequent variant (44%) worldwide and has been previously associated with decreased risk of cerebral hemorrhage and with clinical outcomes in ALL (15). In addition, the *MC2R* rs1893219 A>G variant was associated with better response to glucocorticoid treatment in infantile spasms (23). Herein, the *MC2R* rs1893219 A>G variant was found inversely associated with the risk of childhood ALL in both subtypes (Bcp-ALL and T-ALL), and a gene–environment interplay was observed in children whose mothers had low schooling. Low education is an important risk factor for the mother and the newborn, being associated with infant mortality, less prenatal care, preterm delivery, LBW, and lower breastfeeding (26–28). Since several of these characteristics are associated with maternal stress, they could lead to increased GC levels in the intrauterine environment (29, 30) . Therefore, these potential variations in GC production by the mother together with the potential modulation of MC2R expression levels by its genetic variant could affect the development and the response of the HPA axis in the fetus and modulated ALL risk.

Interestingly, the genetic variant of *GLCCI1*, another gene less explored in the literature, was positively associated with ALL risk. A literature review discloses only 52 articles exploring this gene, and the great majority evaluated the role of *GLICC1* in the treatment response of inflammatory and immunological diseases (31). No studies in leukemias were found, even though GCs are crucial for ALL treatment and relapse prediction (32). The effects of GCs on lymphocytes are translated by the reduction of peripheral circulating lymphocytes through interaction with the GC receptor (GR). Initially, *GlCCI1* was described to bind to specific GRs in the cytoplasm forming complexes and being transferred to the nucleus, thus regulating the transcriptional activity of GC response genes (33). However, recently, Kiuchi and colleagues showed that GLCCI1 is an early marker of apoptosis in murine thymocytes (34), corroborating the study of Tantisira and collaborators (14). In this context, since *GLCCI1* rs37972 C>T leads to lower gene expression, a reduction of apoptosis of inflammatory cells is expected to take place. Thus, based on the biological mechanisms summarized above, we hypothesize that the effect of *GLCCI1* rs37972 C>T in B and T cells in concert with environmental factors could facilitate the expansion of clonal cells of intrauterine origin. This is in accordance with the biological network proposed by Greaves and the adrenal hypothesis proposed by Schmiegelow regarding less fortunate societies and ALL incidence rates (3, 8).

Although the results found here are novel and shed light on the etiopathogenesis of ALL of intrauterine origin, limitations include the lack of knowledge of the functional effects of all genetic variants analyzed as well as the lack of evaluation of known environmental risk factor for ALL development, such as exposure to ionizing radiation and pesticides. Moreover, we encourage further research to explore additional common variants in the HPA axis pathway along with other modulators of the immune response, such as the history of early-life infections.

## 5 Conclusion

Our study has demonstrated a significant association between *MC2R*rs1893219 A>G (protective) and *GLCCI1* rs37972 C>T variants (increased risk) and childhood ALL susceptibility. Based on these lines of evidence, genes controlling the HPA axis activity may play a role in leukemogenesis and further investigation is needed to substantiate our findings.

## Data availability statement

The original contributions presented in the study are included in the article/**Supplementary Material**. Further inquiries can be directed to the corresponding author.

## Ethics statement

The studies involving humans were approved by National Cancer Institute Ethics Committee: CEP/CONEP:1.394.043. The studies were conducted in accordance with the local legislation and institutional requirements. Written informed consent for participation in this study was provided by the participants’ legal guardians/next of kin.

## Author contributions

VMC: conceptualization, methodology, formal analysis, writing the original draft, reviews, and editing. AACF: methodology, statistical analysis and writing the original draft, reviews. PCN: data curation, statistical analysis. FHPB: collaboration, data curation, reviews the manuscript. SCSL: conceptualization, methodology, formal analysis, writing and reviews. MSPO: conceptualization, data curation, writing and editing, funding acquisition. All authors contributed to the article and approved the submitted version.

## References

[1] Enciso-Mora V Hosking FJ Sheridan E Kinsey SE Lightfoot T Roman E. Common genetic variation contributes significantly to the risk of childhood B-cell precursor acute lymphoblastic leukemia. Leukemia (2012) 26:2212–5. doi: 10.1038/leu.2012.89 22456626

[2] Evans T-J Milne E Anderson D de Klerk NH Jamieson SE Talseth-Palmer BA. Confirmation of childhood acute lymphoblastic leukemia variants, ARID5B and IKZF1, and interaction with parental environmental exposures. PloS One (2014) 9:e110255. doi: 10.1371/journal.pone.0110255 25310577 PMC4195717

[3] Greaves M. A causal mechanism for childhood acute lymphoblastic leukaemia. Nat Rev Cancer (2018) 18:471–84. doi: 10.1038/s41568-018-0015-6 PMC6986894 29784935

[4] Swaminathan S Klemm L Park E Papaemmanuil E Ford A Kweon S-M. Mechanisms of clonal evolution in childhood acute lymphoblastic leukemia. Nat Immunol (2015) 16:766–74. doi: 10.1038/ni.3160 PMC4475638 25985233

[5] Schäfer D Olsen M Lähnemann D Stanulla M Slany R Schmiegelow K. Five percent of healthy newborns have an ETV6-RUNX1 fusion as revealed by DNA-based GIPFEL screening. Blood (2018) 131:821–6. doi: 10.1182/blood-2017-09-808402 PMC5909885 29311095

[6] Ford AM Colman S Greaves M. Covert pre-leukaemic clones in healthy co-twins of patients with childhood acute lymphoblastic leukaemia. Leukemia (2023) 37:47–52. doi: 10.1038/s41375-022-01756-1 36536099 PMC9883163

[7] Marcotte EL Ritz B Cockburn M Yu F Heck JE. Exposure to infections and risk of leukemia in young children. Cancer Epidemiol Biomarkers Prev (2014) 23:1195–203. doi: 10.1158/1055-9965.EPI-13-1330 PMC4100471 24793957

[8] Schmiegelow K Vestergaard T Nielsen SM Hjalgrim H. Etiology of common childhood acute lymphoblastic leukemia: the adrenal hypothesis. Leukemia (2008) 22:2137–41. doi: 10.1038/leu.2008.212 18719616

[9] Vogl SE Worda C Egarter C Bieglmayer C Szekeres T Huber J. Mode of delivery is associated with maternal and fetal endocrine stress response. BJOG (2006) 113:441–5. doi: 10.1111/j.1471-0528.2006.00865.x 16489937

[10] Thomas S Izard J Walsh E Batich K Chongsathidkiet P Clarke G. The host microbiome regulates and maintains human health: A primer and perspective for non-microbiologists. Cancer Res (2017) 77:1783–812. doi: 10.1158/0008-5472.CAN-16-2929 PMC5392374 28292977

[11] Heitzer MD Wolf IM Sanchez ER Witchel SF DeFranco DB. Glucocorticoid receptor physiology. Rev Endocr Metab Disord (2007) 8:321–30. doi: 10.1007/s11154-007-9059-8 18049904

[12] Papadimitriou A Priftis KN. Regulation of the hypothalamic-pituitary-adrenal axis. Neuroimmunomodulation (2009) 16:265–71. doi: 10.1159/000216184 19571587

[13] Hu X Deng S Luo L Jiang Y Ge H Yin F. GLCCI1 deficiency induces glucocorticoid resistance *via* the competitive binding of IRF1:GRIP1 and IRF3:GRIP1 in asthma. Front Med (Lausanne) (2021) 8:686493. doi: 10.3389/fmed.2021.686493 34504850 PMC8421568

[14] Tantisira KG Lasky-Su J Harada M Murphy A Litonjua AA Himes BE. Genomewide association between GLCCI1 and response to glucocorticoid therapy in asthma. N Engl J Med (2011) 365:1173–83. doi: 10.1056/NEJMoa0911353 PMC3667396 21991891

[15] Park H-K Chon J Park HJ Chung J-H Baik HH. Association between two promoter polymorphisms (rs1893219 and rs1893220) of MC2R gene and intracerebral hemorrhage in Korean population. Neurosci Lett (2015) 602:1–5. doi: 10.1016/j.neulet.2015.06.032 26115626

[16] Duong-Thi-Ly H Nguyen-Thi-Thu H Nguyen-Hoang L Nguyen-Thi-Bich H Craig TJ Duong-Quy S. Effects of genetic factors to inhaled corticosteroid response in children with asthma: a literature review. J Int Med Res (2017) 45:1818–30. doi: 10.1177/0300060516683877 PMC5805193 29251255

[17] Pombo-de-Oliveira MS EMiLI Study Group Petridou ET Karalexi MA Junqueira MER Braga FHP. The interplay of cesarean-section delivery and first-birth order as risk factors in acute lymphoblastic leukemia. Cancer Epidemiol Biomarkers Prev (2023) 32:371–9. doi: 10.1158/1055-9965.EPI-22-0664 36525650

[18] Infante-Rivard C. Diagnostic x rays, DNA repair genes and childhood acute lymphoblastic leukemia. Health Phys (2003) 85:60–4. doi: 10.1097/00004032-200307000-00012 12852472

[19] Khoury MJ Flanders WD. Nontraditional epidemiologic approaches in the analysis of gene-environment interaction: case-control studies with no controls! Am J Epidemiol (1996) 144:207–13. doi: 10.1093/oxfordjournals.aje.a008915 8686689

[20] Andersson T Alfredsson L Källberg H Zdravkovic S Ahlbom A. Calculating measures of biological interaction. Eur J Epidemiol (2005) 20:575–9. doi: 10.1007/s10654-005-7835-x 16119429

[21] Zadik Z. Adrenal insufficiency in very low birth weight infants. J Pediatr Endocrinol Metab (2010) 23:1–2. doi: 10.1515/JPEM.2010.23.1-2.1 20432799

[22] Flom JD Chiu Y-HM Hsu H-HL Devick KL Brunst KJ Campbell R. Maternal lifetime trauma and birthweight: effect modification by *in utero* cortisol and child sex. J Pediatr (2018) 203:301–8. doi: 10.1016/j.jpeds.2018.07.069 PMC6398337 30197200

[23] Ding Y-X Zou L-P He B Yue W-H Liu Z-L Zhang D. ACTH receptor (MC2R) promoter variants associated with infantile spasms modulate MC2R expression and responsiveness to ACTH. Pharmacogenet Genomics (2010) 20:71–6. doi: 10.1097/FPC.0b013e328333a172 20042918

[24] Barros FC Neto D de LR Villar J Kennedy SH Silveira MF Diaz-Rossello JL. Caesarean sections and the prevalence of preterm and early-term births in Brazil: secondary analyses of national birth registration. BMJ Open (2018) 8:e021538. doi: 10.1136/bmjopen-2018-021538 PMC6078248 30082353

[25] Schneider MO Hübner T Pretscher J Goecke TW Schwitulla J Häberle L. Genetic variants in the glucocorticoid pathway genes and birth weight. Arch Gynecol Obstet (2021) 303:427–34. doi: 10.1007/s00404-020-05761-6 32886236

[26] Gage TB Fang F O’Neill E Dirienzo G. Maternal education, birth weight, and infant mortality in the United States. Demography (2013) 50:615–35. doi: 10.1007/s13524-012-0148-2 PMC3578151 23073749

[27] Ruiz M Goldblatt P Morrison J Kukla L Švancara J Riitta-Järvelin M. Mother’s education and the risk of preterm and small for gestational age birth: a DRIVERS meta-analysis of 12 European cohorts. J Epidemiol Community Health (2015) 69:826–33. doi: 10.1136/jech-2014-205387 PMC4552914 25911693

[28] Laksono AD Wulandari RD Ibad M Kusrini I. The effects of mother’s education on achieving exclusive breastfeeding in Indonesia. BMC Public Health (2021) 21:14. doi: 10.1186/s12889-020-10018-7 33402139 PMC7786474

[29] Wadsby M Nelson N Ingemansson F Samuelsson S Leijon I. Behaviour problems and cortisol levels in very-low-birth-weight children. Nord J Psychiatry (2014) 68:626–32. doi: 10.3109/08039488.2014.907341 24802123

[30] Stoye DQ Boardman JP Osmond C Sullivan G Lamb G Black GS. Saliva cortisol diurnal variation and stress responses in term and preterm infants. Arch Dis Child Fetal Neonatal Ed (2022) 107:558–64. doi: 10.1136/archdischild-2021-321593 PMC9411886 35256524

[31] Feng W Pu W Li J Yuan Y Yan M Yuan S. The GLCCI1 rs37973 variant and the efficacy of inhaled corticosteroids in the treatment of asthma: A meta-analysis. Clin Respir J (2023) 17:568–79. doi: 10.1111/crj.13627 PMC10265153 37157161

[32] Bergeron BP Diedrich JD Zhang Y Barnett KR Dong Q Ferguson DC. Epigenomic profiling of glucocorticoid responses identifies cis-regulatory disruptions impacting steroid resistance in childhood acute lymphoblastic leukemia. Leukemia (2022) 36:2374–83. doi: 10.1038/s41375-022-01685-z PMC9522591 36028659

[33] Hu C-P Xun Q-F Li X-Z Hu X-Y Qin L He R-X. Effects of glucocorticoid-induced transcript 1 gene deficiency on glucocorticoid activation in asthmatic mice. Chin Med J (Engl) (2018) 131:2817–26. doi: 10.4103/0366-6999.246061 PMC6278198 30511684

[34] Kiuchi Z Nishibori Y Kutsuna S Kotani M Hada I Kimura T. GLCCI1 is a novel protector against glucocorticoid-induced apoptosis in T cells. FASEB J (2019) 33:7387–402. doi: 10.1096/fj.201800344RR 30860871

