# Supplementary material for: Interaction between birth characteristics and CRHR1, MC2R, NR3C1, GLCCI1 variants in the childhood lymphoblastic leukemia risk
Source: Front Oncol. 2024 Jan 29;13:1274131. doi: 10.3389/fonc.2023.1274131 (PMC10859751; doi:10.3389/fonc.2023.1274131)
Supplement: Supplementary file 1 [file DataSheet_1.docx]

***Supplementary Material***

**Interaction between mode of delivery, birthweight, *CRHR1*, *MC2R* *NR3C1*, *GLCC1* variants and childhood lymphoblastic leukemia risk**

Vitoria Müller de Carvalho^1^, Alython Araújo Chung Filho^1^, Flávio Henrique Paraguassú Braga^2^, Paulo Chagas Neto^1^, Sheila Coelho Soares Lima^1^, Maria S. Pombo de Oliveira*^1^ per EMiLi Study Group ^3§^ *** Correspondence:** Maria S. Pombo-de-Oliveira, [*mpombo@inca.gov.br*](mailto:mpombo@inca.gov.br)*; masocspoliveira@gmail.com*

# **Supplemental Table 1.** The oligoprimers designed for each gene variant

| **Gene Variant** | **Sequence primer** | **Amplicon size (bp)** |
| --- | --- | --- |
| *CRHR1* rs242941 C>A | F: 5’ GAGGGCCAGGAACCATGAAC 3’ | 146 |
|  | R: 5’ AGTGCTGTTTCCTGAGAGACTG 3’ |  |
| *MC2R* rs1893219 A>G | F: 5’ CGTGGGTGGTCTGGAAAAGA 3’ | 116 |
|  | R: 5’ GACAACACGCTTTGCCTCAG 3’ |  |
| *GLCCI1* rs37972 C>T | F: 5’ CGTGGGTGGTCTGGAAAAGA 3’ | 97 |
|  | R: 5’ CGGGAGCAATTAATGTAAGGATCT 3’ |  |

Abbreviation: bp, base pairs. Designed in: *PrimerBlast Tool ®*, of according Type-it HRM Use guide (Qiagen); F: Forward; R: Reverse.

| Supplemental Table 2. Genotype frequency and the association with ALL risk according to race/ethnicity. Brazil, 2012-2020. | | | | | | | | |
| --- | --- | --- | --- | --- | --- | --- | --- | --- |
|  | White | | |  |  | Multiracial | | |
| Genotype | Case  n (%) | Control  n (%) | OR  (95% CI ) | *P*-value | Case  n (%) | Control  n (%) | OR  (95% CI) | *P*-value |
| *CRHR1*  *r*s242941 C>A | 316 (100) | 143 (100) |  |  | 367 (100) | 236 (100) |  |  |
| CC | 128 (40.5) | 59 (41.3) | 1.0* |  | 136 (37.1) | 77 (32.6) | 1.0* |  |
| CA | 144 (45.6) | 63 (44.1) | 1.05 (0.69-1.62) | 0.81 | 171 (46.6) | 122 (51.7) | 0.79 (0.55-1.14) | 0.21 |
| AA | 44 (13.9) | 21 (14.7) | 0.97 (0.53-1.77) | 0.91 | 60 (16.3) | 37 (15.7) | 0.92 (0.56-1.51) | 0.74 |
| DM |  |  | 1.03 (0.69-1.54) | 0.88 |  |  | 0.82 (0.58-1.16) | 0.27 |
| RM |  |  | 0.94 (0.54-1.65) | 0.83 |  |  | 1.05 (0.67-1.64) | 0.83 |
| *MC2R*  rs1893219 A>G | 298 (100) | 180 (100) |  |  | 310 (100) | 221 (100) |  |  |
| AA | 101 (33.9) | 41 (22.8) | 1.0* |  | 79 (25.5) | 44 (19.9) | 1.0* |  |
| AG | 126 (42.3) | 88 (48.9) | 0.58 (0.37-0.91) | 0.02 | 149 (48.1) | 104 (47.1) | 0.80 (0.51-1.25) | 0.32 |
| GG | 71 (23.8) | 51 (28.3) | 0.57 (0.34-0.94) | 0.03 | 82 (26.5) | 73 (33.0) | 0.63 (0.39-1.02) | 0.06 |
| DM |  |  | 0.58 (0.38-0.88) | 0.01 |  |  | 0.73 (0.48-1.10) | 0.13 |
| RM |  |  | 0.79 (0.52-1.20) | 0.27 |  |  | 0.73 (0.58-1.12) | 0.10 |
| *NR3C1*  rs41423247 G>C | 319 (100) | 175 (100) |  |  | 374 (100) | 232 (100) |  |  |
| GG | 173 (54.2) | 98 (56.0) | 1.0* |  | 237 (63.4) | 146 (62.9) | 1.0* |  |
| GC | 125 (39.2) | 60 (34.3) | 1.18 (0.79-1.75) | 0.41 | 118 (31.6) | 76 (32.8) | 0.96 (0.67-1.36) | 0.81 |
| CC | 21 (6.6) | 17 (9.7) | 0.70 (0.35-1.39) | 0.31 | 19 (5.1) | 10 (4.3) | 1.17 (0.53-2.59) | 0.70 |
| DM |  |  | 1.07 (0.74-1.56) | 0.71 |  |  | 0.98 (0.70-1.38) | 0.91 |
| RM |  |  | 0.65 (0.34-1.28) | 0.21 |  |  | 1.19 (0.54-2.60) | 0.67 |
| *GLCC1*  rs37972 C>T | 310 (100) | 176 (100) |  |  | 368 (100) | 234 (100) |  |  |
| CC | 124 (40.0) | 77 (43.8) | 1.0* |  | 149 (40.5) | 110 (47.0) | 1.0* |  |
| CT | 138 (44.5) | 83 (47.2) | 1.03 (0.70-1.53) | 0.87 | 167 (45.4) | 101 (43.2) | 1.22 (0.86-1.73) | 0.26 |
| TT | 48 (15.5) | 16 (9.1) | 1.86 (0.99-3.51) | 0.05 | 52 (14.1) | 23 (9.8) | 1.67 (0.96-2.89) | 0.07 |
| DM |  |  | 1.17 (0.80-1.70) | 0.42 |  |  | 1.30 (0.94-1.81) | 0.12 |
| RM |  |  | 1.83 (1.01-3.33) | 0.05 |  |  | 1.51 (0.90-2.54) | 0.12 |
|  |  |  |  |  |  |  |  |  |

Abbreviations: n. number; OR, odds ratio; 95% CI confidence interval; RM, recessive model; DM, dominant model.

| Supplemental Table 3. Genotype frequencies and associations with ALL risk according to ALL subtype. Brazil, 2012-2020. | | | | | | | | | | |
| --- | --- | --- | --- | --- | --- | --- | --- | --- | --- | --- |
|  | Bcp-ALL | | | |  |  | T-ALL | | | |
| Genotype | Case  n (%) | Control  n (%) | OR  (95% CI) | Adj OR (IC95%) | *P*-value | Case  n (%) | Control  n (%) | OR  (95% CI) | Adj OR  (95% CI) | *P*-value |
| *CRHR1*  *r*s242941 C>A | 587 (100) | 379 (100) |  |  |  | 96 (100) | 379 (100) |  |  |  |
| CC | 227 (38.7) | 136 (35.9) | 1.0* | 1.0* |  | 37 (38.5) | 136 (35.9) | 1.0* | 1.0* |  |
| CA | 276 (47) | 185 (48.8) | 0.89 (0.67-1.19) | 0.92 (0.69-1.22) | 0.55 | 39 (40.6) | 185 (48.8) | 0.77 (0.47-1.28) | 0.79 (0.48-1.30) | 0.35 |
| AA | 84 (14.3) | 58 (15.3) | 0.87 (0.58-1.29) | 0.88 (0.59-1.31) | 0.53 | 20 (20.8) | 58 (15.3) | 1.27 (0.68-2.37) | 1.22 (0.65-2.29) | 0.54 |
| DM |  |  | 0.89 (0.68-1.16) | 0.91 (0.69-1.19) | 0.49 |  |  | 0.89 (0.56-1.42) | 0.90 (0.57-1.43) | 0.65 |
| RM |  |  | 0.92 (0.64-1.33) | 0.93 (0.64-1.33) | 0.68 |  |  | 1.46 (0.83-2.57) | 1.47 (0.83-2.60) | 0.18 |
| *MC2R*  rs1893219 A>G | 527 (100) | 401 (100) |  |  |  | 81 (100) | 401 (100) |  |  |  |
| AA | 149 (28.3) | 85 (21.2) | 1.0* | 1.0* |  | 31 (38.3) | 85 (21.2) | 1.0* | 1.0* |  |
| AG | 242 (45.9) | 192 (47.9) | 0.72 (0.52-1.00) | 0.73 (0.53-1.01) | 0.06 | 33 (40.7) | 192 (47.9) | 0.47 (0.27-0.82) | 0.46 (0.26-0.80) | 0.01 |
| GG | 136 (25.8) | 124 (30.9) | 0.63 (0.44-0.90) | 0.64 (0.45-0.92) | 0.02 | 17 (21.0) | 124 (30.9) | 0.38 (0.20-0.72) | 0.38 (0.20-0.73) | <0.01 |
| DM |  |  | 0.68 (0.50-0.93) | 0.69 (0.51-0.94) | 0.02 |  |  | 0.43 (0.26-0.72) | 0.43 (0.26-0.72) | <0.01 |
| RM |  |  | 0.78 (0.58-1.04) | 0.79 (0.59-1.05) | 0.10 |  |  | 0.59 (0.33-1.06) | 0.59 (0.33-1.05) | 0.07 |
| *NR3C1*  rs41423247 G>C | 601 (100) | 407 (100) |  |  |  | 92 (100) | 407 (100) |  |  |  |
| GG | 358 (59.6) | 244 (60.0) | 1.0* | 1.0* |  | 52 (56.5) | 244 (60.0) | 1.0* | 1.0* |  |
| GC | 208 (34.6) | 136 (33.4) | 1.04 (0.80-1.37) | 1.03 (0.78-1.35) | 0.84 | 35 (38.0) | 136 (33.4) | 1.21 (0.75-1.95) | 1.21 (0.75-1.96) | 0.42 |
| CC | 35 (5.8) | 27 (6.6) | 0.88 (0.52-1.50) | 0.87 (0.51-1.48) | 0.60 | 5 (5.4) | 27 (6.6) | 0.87 (0.32-2.36) | 0.93 (0.34-2.54) | 0.88 |
| DM |  |  | 1.02 (0.79-1.31) | 1.00 (0.77-1.30) | 0.99 |  |  | 1.15 (0.73-1.82) | 1.17 (0.74-1.85) | 0.50 |
| RM |  |  | 0.87 (0.52-1.46) | 0.85 (0.50-1.43) | 0.53 |  |  | 0.81 (0.30-2.16) | 0.83 (0.31-2.23) | 0.72 |
| *GLCC1*  rs37972 C>T | 581 (100) | 410 (100) |  |  |  | 97 (100) | 410 (100) |  |  |  |
| CC | 232 (39.9) | 187 (45.6) | 1.0* | 1.0* |  | 41 (42.3) | 187 (45.6) | 1.0* | 1.0* |  |
| CT | 266 (45.8) | 184 (44.9) | 1.17 (0.89-1.53) | 1.16 (0.89-1.52) | 0.27 | 39 (40.2) | 184 (44.9) | 0.97 (0.60-1.57) | 0.97 (0.60-1.58) | 0.91 |
| TT | 83 (14.3) | 39 (9.5) | 1.72 (1.12-2.63) | 1.71 (1.11-2.61) | 0.01 | 17 (17.5) | 39 (9.5) | 1.99 (1.03-3.86) | 1.99 (1.02-3.85) | 0.04 |
| DM |  |  | 1.26 (0.98-1.63) | 1.26 (0.97-1.63) | 0.08 |  |  | 1.15 (0.73-1.79) | 1.15 (0.74-1.80) | 0.54 |
| RM |  |  | 1.59 (1.06-2.37) | 1.58 (1.06-2.37) | 0.03 |  |  | 2.02 (1.09-3.75) | 2.01 (1.08-3.74) | 0.03 |
| Abbreviations: n, number; OR, odds ratio; 95% CI, confidence interval; RM, recessive model; DM, dominant model; AdjOR, adjusted odd ratio by race. | | | | | | | | | | |

| Supplemental Table 4. Genotype frequency and the associations with ALL risk according to sex. Brazil, 2012-2020. | | | | | | | | | | |
| --- | --- | --- | --- | --- | --- | --- | --- | --- | --- | --- |
|  | Male | | | |  |  | Female | | | |
| Genotype | Case  n (%) | Control  n (%) | OR  (95% CI) | Adj OR  (95% CI) | *P*-value | Case  n (%) | Control  n (%) | OR  (95% CI) | Adj OR  (95%CI) | *P*-value |
| *CRHR1*  *r*s242941 C>A | 385 (100) | 379 (100) |  |  |  | 298 (100) | 379 (100) |  |  |  |
| CC | 152 (39.5) | 136 (35.9) | 1.0* | 1.0* |  | 112 (37.6) | 136 (35.9) | 1.0* | 1.0* |  |
| CA | 178 (46.2) | 185 (48.8) | 0.86 (0.63-1.17) | 0.87 (0.64-1.19) | 0.40 | 137 (46) | 185 (48.8) | 0.90 (0.64-1.26) | 0.93 (0.66-1.30) | 0.67 |
| AA | 55 (14.3) | 58 (15.3) | 0.85 (0.55-1.31) | 0.85 (0.55-1.32) | 0.48 | 49 (16.4) | 58 (15.3) | 1.03 (0.65-1.62) | 1.05 (0.66-1.66) | 0.84 |
| DM |  |  | 0.86 (0.64-1.15) | 0.87 (0.65-1.17) | 0.36 |  |  | 0.93 (0.68-1.27) | 0.96 (0.70-1.32) | 0.80 |
| RM |  |  | 0.92 (0.62-1.38) | 0.93 (0.62-1.39) | 0.73 |  |  | 1.09 (0.72-1.65) | 1.10 (0.73-1.67) | 0.65 |
| *MC2R*  rs1893219 A>G | 342 (100) | 401 (100) |  |  |  | 266 (100) | 401 (100) |  |  |  |
| AA | 108 (31.6) | 85 (21.2) | 1.0* | 1.0* |  | 72 (27.1) | 85 (21.2) | 1.0* | 1.0* |  |
| AG | 151 (44.2) | 192 (47.9) | 0.62 (0.43-0.88) | 0.62 (0.43-0.88) | 0.01 | 124 (46.6) | 192 (47.9) | 0.76 (0.52-1.12) | 0.79 (0.53-1.16) | 0.23 |
| GG | 83 (24.3) | 124 (30.9) | 0.53 (0.35-0.78) | 0.53 (0.36-0.79) | <0.01 | 70 (26.3) | 124 (30.9) | 0.67 (0.43-1.02) | 0.71 (0.46-1.09) | 0.12 |
| DM |  |  | 0.58 (0.42-0.81) | 0.58 (0.42-0.81) | <0.01 |  |  | 0.72 (0.51-1.04) | 0.75 (0.52-1.08) | 0.12 |
| RM |  |  | 0.72 (0.52-0.99) | 0.72 (0.52-0.99) | 0.04 |  |  | 0.80 (0.57-1.13) | 0.82 (0.58-1.16) | 0.25 |
| *NR3C1*  rs41423247 G>C | 398 (100) | 407 (100) |  |  |  | 295 (100) | 407 (100) |  |  |  |
| GG | 244 (61.3) | 244 (60.0) | 1.0* | 1.0* |  | 166 (56.3) | 244 (60.0) | 1.0* | 1.0* |  |
| GC | 130 (32.7) | 136 (33.4) | 0.96 (0.71-1.29) | 0.95 (0.71-1.29) | 0.75 | 113 (38.3) | 136 (33.4) | 1.22 (0.89-1.68) | 1.20 (0.87-1.65) | 0.26 |
| CC | 24 (6.0) | 27 (6.6) | 0.89 (0.50-1.58) | 0.89 (0.50-1.60) | 0.70 | 16 (5.4) | 27 (6.6) | 0.87 (0.46-1.67) | 0.86 (0.45-1.65) | 0.66 |
| DM |  |  | 0.94 (0.71-1.25) | 0.94 (0.71-1.25) | 0.67 |  |  | 1.16 (0.86-1.58) | 1.15 (0.85-1.56) | 0.37 |
| RM |  |  | 0.90 (0.51-2.16) | 0.89 (0.50-1.58) | 0.69 |  |  | 0.81(0.43-1.53) | 0.80 (0.42-1.51) | 0.48 |
| *GLCC1*  rs37972 C>T | 378 (100) | 410 (100) |  |  |  | 300 (100) | 410 (100) |  |  |  |
| CC | 151 (39.9) | 187 (45.6) | 1.0* | 1.0* |  | 122 (40.7) | 187 (45.6) | 1.0* | 1.0* |  |
| CT | 164 (43.4) | 184 (44.9) | 1.10 (0.82-1.49) | 1.11 (0.82-1.49) | 0.51 | 141 (47) | 184 (44.9) | 1.17 (0.86-1.61) | 1.17 (0.85-1.61) | 0.33 |
| TT | 63 (16.7) | 39 (9.5) | 2.00 (1.27-3.15) | 2.00 (1.27-3.14) | <0.01 | 37 (12.3) | 39 (9.5) | 1.45 (0.88-2.41) | 1.46 (0.88-2.42) | 0.14 |
| DM |  |  | 1.26 (0.95-1.67) | 1.26 (0.95-1.67) | 0.11 |  |  | 1.22 (0.91-1.65) | 1.22 (0.90-1.65) | 0.20 |
| RM |  |  | 1.90 (1.24-2.91) | 1.90 (1.24-2.92) | <0.01 |  |  | 1.24 (0.83-2.16) | 1.34 (0.83-2.17) | 0.23 |
| Abbreviations: n, number; OR, odds ratio; 95% CI, 95% confidence interval; R, recessive model; DM, dominant model. Adj. OR, adjusted odd ratio by race. | | | | | | | | | | |
